# Outcome of Congenitally Hypothyroid Screening Program in Isfahan: Iran From Prevention to Treatment

**Published:** 2010

**Authors:** Mahin Hashemipour, Elham Hashemi Dehkordi, Silva Hovsepian, Massoud Amini, Leila Hosseiny

**Affiliations:** 1 Professor of Pediatric Endocrinology, Department of Pediatrics, Isfahan Endocrine & Metabolism Research Center (IEMRC), Isfahan University of Medical Sciences (IUMS), Isfahan, Iran; 2 Pediatrician, Department of Pediatrics, IEMRC, IUMS, Isfahan, Iran; 3 General Practitioner, Research Assistant, IEMRC, Isfahan, Iran; 4 Professor of Endocrinology, Department of Internal Medicine, IEMRC, IUMS, Isfahan, Iran; 5 Coordinator of Isfahan Congenital Hypothyroidism Screening Program, Isfahan Health Center, IUMS, Isfahan, Iran

**Keywords:** Congenital hypothyroidism, Treatment, Isfahan, Iran

## Abstract

**Objectives:**

Early and proper treatment is crucial to prevent neuropsychologic deficits in congenital hypothyroidism (CH). Considering the high prevalence of CH in Isfahan, the aim of this study was to evaluate the outcome of treatment in CH patients.

**Methods:**

In this study CH neonates diagnosed during screening program in Isfahan from May 2002 to September 2009 were studied. Frequent visits were performed to CH patients to monitor and follow their treatments. Quality of treatment was assessed by evaluating mean age of treatment initiation and mean TSH and T4 levels before and after treatment and during the first and second years according to their normal reference ranges.

**Results:**

Of 225,224 screened neonates, 536 were diagnosed as CH patients. The prevalence of CH was 1/420 live births. Mean age at starting treatment was 22.9 ± 13.2 days. In 93.7% of patients, treatment was begun before the 45th day of life. In the first measurement after initiating the treatment, T4 and TSH were not in their acceptable range in 3.9% and 9.8% of CH patients, respectively. Mean T4 and TSH reached to normal range during the treatment period. T4 reached the normal range earlier than TSH.

**Conclusions:**

The mean age of treatment initiation was in acceptable range but the findings suggest that both early and high-dose treatments are crucial for optimal treatment, especially in patients with severe CH. Further studies are needed to determine the outcome of treatment specially regarding to different etiologies of CH.

## INTRODUCTION

Thyroid hormone is important for human development, especially the central nervous system^1^,^2^ and deficiency of this hormone during the first years after birth results in a spectrum of neuropsychological disorders.^3^–^5^ Therefore, early detection and treatment are crucial to prevent mental retardation in congenital hypothyroidism. Moreover, proper and adequate initial dosage of levothyroxine is essential for prevention of the adverse neurodevelopmental consequences of CH.^6^ During the past decades, newborns screening for CH has become an important activity in most developed and some developing countries and it is one of the great successes of preventive medicine for elimination of mental retardation related to CH. Before the advent of screening in 1970, one third of children with congenital hypothyroidism were not detected until after their third month of life, at which point they had developed irreversible mental retardation.^7^–^9^ Five or six month delay in treatment of CH could result in an IQ of 70 in a child who probably otherwise would have had normal intelligence.^10^ Prevalence of CH in Iran, have reported to be 1/1433 in Fars province, 1/914 in Tehran and 1:748 in Isfahan.^11^–^13^

Screening program of CH in Isfahan, which is located in the central part of Iran began in 2002.^14^ Considering that evaluating the outcome of screening and especially quality of the treatment seems to be essential for planning more appropriate screening programs and lack of this kind of study in Iran inspite of starting the CH screening program in the last decade in Iran, the aim of this study was to report the findings related to the treatment of CH patients in Isfahan.

## METHODS

In this cross-sectional study, newborns that were referred to Isfahan Endocrine and Metabolism Research Center for treatment and follow up during CH screening program in Isfahan, from May 2002 to September 2009 were studied. From May 2002 till April 2005, T4 and TSH serum concentrations of all 3-7 dayold newborns, born in all 17 hospitals of the city (of Isfahan), were measured by radioimmunoassay (RIA) and immunoradiometric assay (IRMA), respectively, using Kavoshyar (Iran-Tehran) kits. Thyroid function tests were performed by Berthold-LB2111 unit gamma counter equipment. Newborns with abnormal screening results were re-examined and those with abnormal T4 and TSH level on their second measurement were diagnosed as CH patients and received treatment and regular follow up.^10^ After implementation of nationwide CH screening program in Iran in April 2005, screening was performed using filter paper. Neonates with TSH>10 were recalled and those with abnormal T4 and TSH levels on their second measurements were diagnosed as CH patient and received treatment and regular follow up. Levothyroxine was prescribed for hypothyroid neonates at a dose of 10-15 mg/kg/day as soon as the diagnosis was confirmed. Monitoring of TSH and T4 was done every 1-2 months during the first year of life and every 1-3 months during the second and third years. Frequent visits were performed to CH patients to mention appropriate T4 and TSH concentrations.^15^ The goal of treatment was to keep T4 in the upper half of normal range [7.2-15.6 μg/dl (11.2) for 1-12 month-old and 7.3-15.0 (10.5) for 1-5 year-old children) and the TSH around 1 mIU/L (ranged 0.6-6.3). Levothyroxine dosage was adjusted with growing the CH patients.^16^–^19^ Treatment considered acceptable and optimal if began between 14^th^ and 45^th^ days of life.^14^ Quality of treatment was assessed by evaluating the mean age of treatment initiation, and the mean TSH and T4 levels before and after treatment and during the first and second years of life. Cases with unacceptable T4 and TSH levels were evaluated. Neonates with CH were recommended to do thyroid scintigraphy before treatment.

### Statistical analysis

Mean and standard deviation of T4 and TSH levels in neonates were estimated using SPSS version 13. Mean levels of studied variables were compared using repeated measure ANO-VA test. P<0.05 was considered statistically significant.

## RESULTS

**Figure 1:**
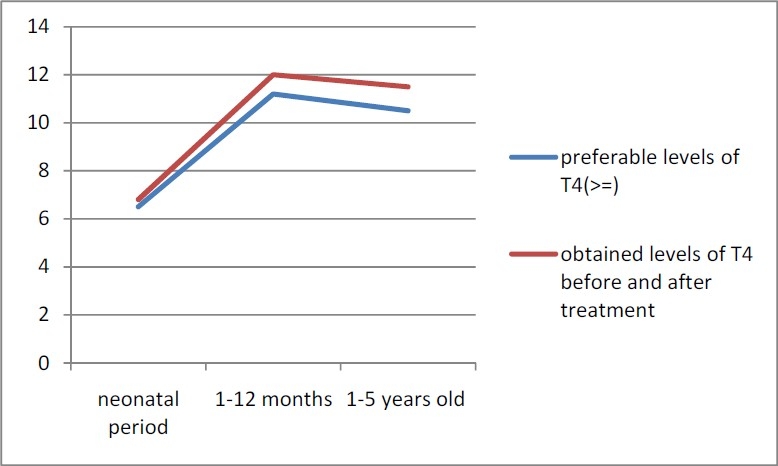
Preferable and obtained levels of T4 before and after treatment

**Figure 2:**
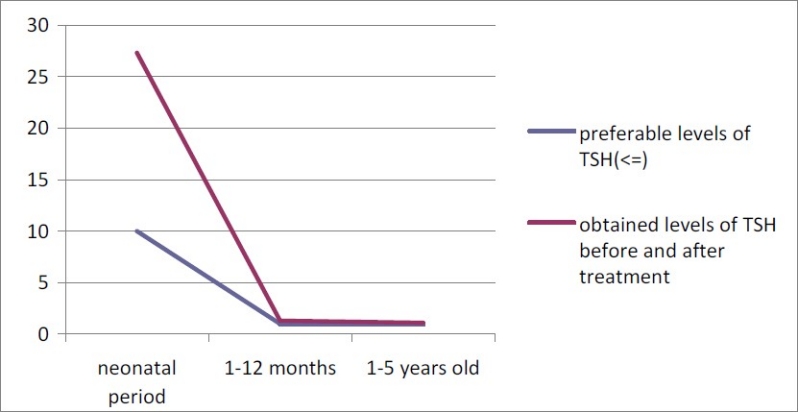
Preferable and obtained levels of TSH before and after treatment

During this study 225,224 neonates were screened for CH. The coverage percent in our study was 96.6%. Overall, 1.7% of screened neonates were recalled and 536 of them were diagnosed as congenitally hypothyroid neonates (both permanent and transient) and were undergone treatment. The overall prevalence of CH was 1 in 420 live births. The mean and median T4 and TSH levels before and after initiation of treatment, the last ones and the mean TSH and T4 levels during the first and second years of life are presented in Table 1 ; 28.5% of our hypothyroid patients were 0-1 year-old and 18.5% were 1-2 year-old and 53% was older than 2 years. Before treatment, 33.3%, 66.7% and 90.8% of studied newborns had TSH level >40 mIU/L, >20 and >10 mIU/L, respectively. In the first measurement after initiating the treatment, 3.9% of CH patients had T4<7.2 mg/dl (lower limit of normal T4 for 1-12 month-old children) and 26% had T4<11.2 μg/dl (the upper half of the normal range); 90.2% of hypothyroid subjects had TSH<6.3 mIU/L (upper limit of TSH for 1-12 month-old children) and 75.4% had mean TSH ranged 0.6-2.1 mIU/L according to the normal range of TSH for 1-12 month-old children. Preferable and obtained levels f T4 and TSH (median) before and after treatment are presented in Figures 1 and 2. Patients with lower T4 and higher TSH levels than the accepted normal range had interrupted their treatments for a period of time without counseling with the physicians, therefore T4 and TSH become lower and higher, respectively because of poor coordination. During the first year of treatment, 96.9% and 76% had T4>7.2 μg/dl and >11.2 μg/dl, respectively; 91% had TSH<6.3 mIU/L and 76.7% had TSH<2.1 mIU/L. During the second year of life, 96.2% and 78.5% had T4>7.3 μg/dl and >10.5 μg/dl (upper half of the normal range); 95.7% had TSH<6.3 mIU/L and 84.9% had TSH<2 mIU/L. After treatment, according to the level of TSH of the last measurement, 2.9%, 6.1% and 11.4% of studied newborns had TSH>40 mIU/L, >20 and TSH>10, respectively; 88.6% of them had TSH<10. Mean age of starting treatment was 22.9 ± 13.2 days. In 93.7% of patients, treatment was begun before the 45^th^ day of life and in 26.1% it was begun during the first 14 days of life. The mean dose of levothyroxine was 41.2 ± 18.3 μg.

**Table 1:** Mean (median) T4 and TSH levels before and during treatment of congenitally hypothyroid patients, Isfahan, Iran

	T4 (μg/dl)	TSH (mIU/L)
Before treatment	7.9 ± 10.4 (6.8)	45.6 ± 53.7 (27.1)
After initiation of treatment	11.2 ± 8.8 (10.8)	25.1 ± 46.3 (10.9)
During the first year of life	12.2 ± 8.4 (12.0)	14.4 ± 33.3 (1.3)
During the second year of life	11.8 ± 4.9 (11.5)	3.1 ± 9.6 (1.1)
The last measurement	12.1 ± 21.6 (11.5)	2.6 ± 4.5 (1.2)

## DISCUSSION

During this study, 225,224 newborns were screened on their 3^rd^ -7^th^ day of life. The coverage percent of our study was 96.6%. The overall prevalence of CH was 1 in 420 live births (both transient and permanent), which was higher than those in other studies, both in Iran and other countries.^11^,^12^,^20^ CH occurs approximately in 1/3000-1/4000 live births in most developed countries^21^, so the overall prevalence of CH in this study was approximately 7.1-9.5 times greater than that reported from mentioned countries. It may be due to different ethnic, environmental, genetic and autoimmune factors.^22^–^25^ According to some previous studies in this field, parental consanguinity may be considered as one of the main reasons for this high prevalence; however, further studies are needed for more accurate conclusion.^26^ According to many studies early treatment with thyroxine might prevent the adverse neurodevelopmental consequences of delayed diagnosis and treatment.^27^,^28^ Therefore, age at the start of treatment has been demonstrated to be an important determinant of neurodevelopmental outcome^29^, and screening programs aim to reduce the postnatal period of hypothyroidism as much as possible. On the other hand, there is disagreement on whether early treatment with thyroxine fully protects infants against neurological damage.^30^ Some studies have shown that an earlier onset of treatment in CH patients is correlated with better subsequent intellectual development^31^–^33^ but, others have not reported this correlation.^34^,^35^ Many follow-up studies have demonstrated that even with more optimal treatment, CH patients do still experience some developmental delay. However, in most countries treatment is now started earlier, usually being initiated within the first 2 weeks of life^36^ and the accepted range for starting the treatment is considered the 45^th^ day of life.^15^ In our study, mean age of treatment was 22.9 ± 13.2 days that was in acceptable range. In the study of Ordookhani et al., treatment was initiated at 11 ± 5 days of life.^12^ Mean age of treatment was 17 days in the screening program of Wales by heel-Prick method.^37^ It was also 17 days in another study in England^29^ and 10.3 days in the screening program of Saudi Arabia which was considered as the real measure of success in that project.^38^ In a study in France^39^ that investigated the influence of severity of CH and the adequacy of treatment in school achievement in young adolescents, the mean age of treatment was similar to our study (22.8 ± 6.8 days). These differences are the result of different screening methods. In studies using cord blood sample for screening, the mean age of starting treatment was earlier. According to the study of Bakker et al.^40^ in which the dynamics of the plasma concentrations of TSH, FT4 and T4 following thyroxine supplementation was investigated in CH patients, plasma T3 and T4 concentrations reached the normal range a few days after thyroxine treatment was started while, normalization of plasma TSH concentration took several weeks. In our study, median T4 level was in normal range after initiation of treatment but median TSH level was not, at that time. During the follow up period and also during the first year of life, the median levels of both T4 and TSH were in normal range. Therefore, the findings of our study were similar to the results of Bakker et al. study. In the first measurement after initiating the treatment, T4 and TSH were not in their acceptable range in 3.9% and 9.8% of CH patients, respectively. It was because that parents of mentioned patients did not refer their children regularly for follow up treatments or discontinued treatments without counseling with physicians. Inspite of the fact that normalization of TSH occurs later than that of T4, these findings suggest that both early and high-dose treatments are crucial for optimal treatment. However, recent studies recommended a higher levothyroxine starting dose of 12-17 microg/kg/d, especially in patients with severe CH. By using this recommendation, T4 will normalize in 3 days and TSH in 2 weeks.^41^ Our study had some limitations. We didn’t study the relation between different etiologies and severity of CH with the outcome of treatment. Therefore, further studies are needed to investigate the outcome of treatment according to different etiologies and severity of CH and its relation with neurodevelopmental outcomes in CH patients. In sum, in accordance with screening programs, studies in the field of optimal follow up and treatment-adequacy issues will provide more information for more effective screening programs.
